# Microglial activation in the hippocampus of hypercholesterolemic rabbits occurs independent of increased amyloid production

**DOI:** 10.1186/1742-2094-4-20

**Published:** 2007-08-24

**Authors:** Qing-Shan Xue, D Larry Sparks, Wolfgang J Streit

**Affiliations:** 1 Department of Neuroscience, University of Florida College of Medicine and McKnight Brain Institute, 100 Newell Drive, Gainesville FL 32611, USA; 2 Roberts Laboratory for Neurodegenerative Disease Research, Sun Health Research Institute, Sun City, AZ, USA

## Abstract

**Background:**

Rabbits maintained on high-cholesterol diets are known to show increased immunoreactivity for amyloid beta protein in cortex and hippocampus, an effect that is amplified by presence of copper in the drinking water. Hypercholesterolemic rabbits also develop sporadic neuroinflammatory changes. The purpose of this study was to survey microglial activation in rabbits fed cholesterol in the presence or absence of copper or other metal ions, such as zinc and aluminum.

**Methods:**

Vibratome sections of the rabbit hippocampus and overlying cerebral cortex were examined for microglial activation using histochemistry with isolectin B_4 _from *Griffonia simplicifolia*. Animals were scored as showing either focal or diffuse microglial activation with or without presence of rod cells.

**Results:**

Approximately one quarter of all rabbits fed high-cholesterol diets showed evidence of microglial activation, which was always present in the hippocampus and not in the cortex. Microglial activation was not correlated spatially with increased amyloid immunoreactivity or with neurodegenerative changes and was most pronounced in hypercholesterolemic animals whose drinking water had been supplemented with either copper or zinc. Controls maintained on normal chow were largely devoid of neuroinflammatory changes, but revealed minimal microglial activation in one case.

**Conclusion:**

Because the increase in intraneuronal amyloid immunoreactivity that results from administration of cholesterol occurs in both cerebral cortex and hippocampus, we deduce that the microglial activation reported here, which is limited to the hippocampus, occurs independent of amyloid accumulation. Furthermore, since neuroinflammation occurred in the absence of detectable neurodegenerative changes, and was also not accompanied by increased astrogliosis, we conclude that microglial activation occurs because of metabolic or biochemical derangements that are influenced by dietary factors.

## Background

A number of neuropathological changes similar to those characteristically associated with Alzheimer's disease have been reported in hypercholesterolemic rabbits, and thus the cholesterol-fed rabbit offers a pertinent animal model for investigating some of the mechanisms that underlie disease pathogenesis [1,2]. Perhaps most relevant is the fact that addition of cholesterol to the diet consistently results in increased immunoreactivity for amyloid beta protein within neurons of the cerebral and hippocampal cortices of these animals [3,4]. Inflammatory changes, such as microglial activation and leukocyte extravasation, have also been reported in cholesterol-fed rabbits, but unlike the enhanced accumulation of amyloid neuroinflammatory changes are not found uniformly in all hypercholesterolemic animals [5]. When neuroinflammation does occur it tends to be limited affecting relatively small areas rather than an entire region. In the past, we have assumed that the inciting stimulus for neuroinflammation is provided by the increase in amyloid beta protein that results from high serum cholesterol levels. This assumption seemed reasonable in light of large numbers of studies reporting proinflammatory effects of amyloid beta peptides over many years [6-14].

The finding that addition of small amounts of copper to the drinking water of cholesterol-fed rabbits amplifies the accumulation of intraneuronal amyloid in cortex and hippocampus and leads to cognitive dysfunction [15] has prompted us to reexamine brains from animals treated in this fashion for neuroinflammatory changes. Our expectation was that concomitant with the enhanced accumulation of amyloid there would be increased neuroinflammation. At the same time, since zinc-supplemented drinking water does not have a significant effect on amyloid accumulation [16], we expected to see no change in neuroinflammation in rabbits receiving zinc. However, contrary to this hypothesis our current findings now show that animals from both copper and zinc-supplemented groups show similar levels of microglial activation. In addition, microglial activation in all animals maintained on cholesterol diets, regardless of metals added, was confined to the hippocampal region. This leads us to think that microglial activation in the cholesterol-fed rabbit is unrelated to intraneuronal amyloid accumulation, but is triggered instead by metabolic or biochemical abnormalities in the hippocampus caused by elevated serum cholesterol levels.

## Methods

### New Zealand white rabbits

Adolescent male New Zealand white rabbits (3000–4000 g) were housed in the rabbit facility at SHRI operating under the guidelines of the USDA with a 12:12 light cycle, at 67 ± 7°F, and 45–50% humidity. Animals were randomly assigned to one of seven groups as a subset of a larger IACUC approved experimental protocol. Some animals received normal chow and allowed either distilled water or distilled water with 0.12 PPM copper added (n = 8) *ad libitum*. Other animals were administered 2% cholesterol diet and allowed tap water (n = 4) or distilled water (n = 4), or distilled water with 0.12 PPM copper ion (as sulfate, n = 4), 0.36 PPM zinc (as sulfate, n = 5) or 0.36 PPM aluminum (as sulfate, n = 5) *ad libitum*. Control and cholesterol diets were commercially obtained from Purina Mills, Inc. (Laboratory Rabbit Diet with and without 2% cholesterol) and were administered for 10 weeks. Dietary food intake was limited to one cup per day (8 oz) and *ad libitum *water consumption varied between 32 and 40 oz/day. The animal protocol (# 0403) was approved by the Sun Health Research Institute Institutional Animal Care and Use Committee.

### Water analysis

Water was analyzed by US Filters (Vivendi Environment), an EPA Certified Water Quality Testing Laboratory for levels of Arsenic (EPA 200.9), Mercury (EPA 245.1), and organics (total organic carbo-TOC; SM5310C) as special studies, and for a 'Standard A' assessment (EPA 200.7, EPA 300.0) to include levels of aluminum, calcium, magnesium, sodium, potassium, barium, strontium, iron, copper, manganese, zinc, chloride, sulfate, nitrate, fluoride, and silica.

### Tissue processing

Animals in each group were sacrificed ten weeks after initiating the experimental dietary (food and water) protocol. On the day of sacrifice, animals were administered a cocktail of Ketamine and Xylazine (IM; 45–75 mg/kg and 5–10 mg/kg respectively). Anesthetized animals were secured to a stainless steel surgical apparatus, the heart was exposed and a butterfly needle was inserted in the left apex, and blood was collected in purple top (EDTA) vacutainer tubes for chemical analysis. Thereafter, a needle attached to the perfusion apparatus was inserted and secured in the left apex of the heart, the vena cava was incised and perfusion was initiated. Animals were perfused under pressure with 120 ml of 4% paraformaldehyde at a constant rate of 5 ml/min using a constant pressure pump. A full necropsy was performed on each animal. Fifty-micron vibratome sections of hippocampus and hippocampal cortex of the brain were prepared for subsequent staining.

### Lectin histochemistry

Microglial cells were visualized in brain sections using lectin binding, as described [17,18]. Following a rinse in PBS, sections were incubated in lectin GSA I-B_4_-HRP (Sigma Chemical Co., L5391), diluted to 5 μg/ml in 0.1% Triton/PBS overnight at 4°C. After washing with PBS, lectin binding sites were visualized with 3,3'-diabimobenzidine (DAB)-H_2_O_2 _substrate. All sections were dehydrated through ascending alcohols, cleared in xylenes and coverslipped with Permount. Selected sections were counterstained with 0.5% cresyl violet.

### Double fluorescent labeling of microglia and astrocytes

In order to determine if microglial activation was accompanied by astrogliosis, double-labeling for both glial cell types was performed. Sections were rinsed in PBS, followed by blockage of non-specific binding of antibodies in 10% normal goat serum in PBS for 1 hr at 37°C. Sections were then incubated in a mixed solution of rabbit polyclonal anti-glial fibrillary acidic protein (GFAP, DakoCytomation, Denmark A/S, diluted at 1:200) and biotinylated isolectin B_4 _(5 μg/ml, Sigma, L2140) in 5% goat serum with 0.1% Triton X-100 in PBS at 4°C for overnight. After three washes with PBS, sections were incubated in a mixed solution of highly cross-adsorbed goat anti-rabbit IgG conjugated with Alexa fluor 488 (Molecular Probes, A11034, diluted at 1:300) and avidin conjugated with Alexa fluor 594 (Invitrogen, S32356, diluted at 1:500) in 5% goat serum with 0.1% Triton X-100 in PBS for 1 hr at room temperature. Following three washes, sections were mounted onto glass slides and coverslipped with GEL/MOUNT (Biomeda corp., Foster City, CA).

### Immunolabeling for ubiquitin

In order to detect ubiquitinated neurons or neurites indicative of neurodegeneration, single staining was performed using a monoclonal antibody against ubiquitin (hybridoma supernatant provided by Dr. Gerry Shaw [19]). Binding sites were visualized using biotinylated goat anti-mouse IgG antibodies (Vector Laboratories, Cat. No. BA-9200), amplified by avidin conjugated with HRP, and DAB-H_2_O_2 _substratum. Ubiquitin immunolabeling was also performed with goat anti-mouse IgG conjugated with Alexa fluor 488 (Molecular Probes, A11029, diluted at 1:500).

### Observation and imaging

Slides were examined with a Zeiss Axioskop 2 microscope. Digital images were captured with a Spot RT3 digital camera (Diagnostic Instruments Inc.; Sterling Heights, MI). For double fluorescence labeling, images were originally captured in black and white. Images were pseudo-colored and/or digitally merged from images captured at single fluorochrome using Adobe Photoshop software (Adobe Systems Inc.; San Jose, CA).

## Results

**Figure 1:**
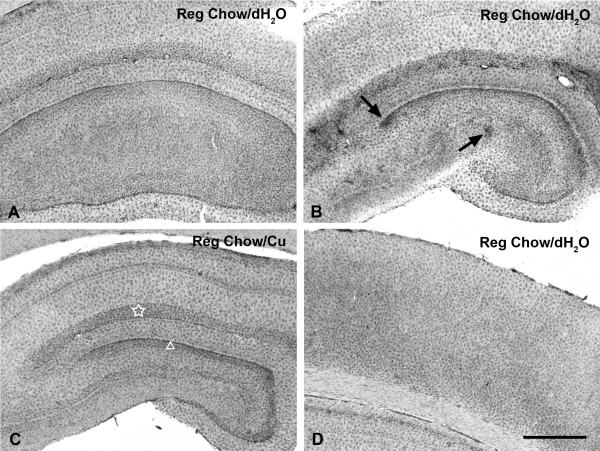
Lectin staining for microglia in the hippocampus (**A–C**) and cerebral cortex (**D**) of control rabbits receiving regular chow. **A, C, D, **microglia are distributed evenly throughout the parenchyma as resting cells showing slightly greater density in the subgranular zone of the dentate gyrus (triangle in **C**) and the stratum lacunosum moleculare (star in **C**). One of the control animals shows small foci of minimal microglial activation (arrows in **B**). Scale bar: 1,000 μm

Similar to our prior observations regarding microgliosis in hypercholesterolemic rabbits [5], the current results revealed considerable variability in neuroinflammation among animals in any given group of animals fed a cholesterol-containing diet. Most animals on cholesterol diets did not show any signs of microglial activation while some showed focal and/or diffuse patterns of activation, as detailed below. Controls maintained on regular chow and dH_2_O with or without copper ion added showed no evidence of microglial activation in 7 out of 8 animals (Table 1, Figs. 1A,C,D). However, in one case we were able to observe small foci of enhanced staining intensity in the dentate gyrus indicative of low level activation (Fig. 1B). For purposes of scoring and comparing the intensity of microglial activation in individual animals, we designated the pattern observed in this control animal as "minimal" neuroinflammation.

**Table 1:** Qualitative assessment of microglial activation in hippocampi of rabbits fed different diets.

Animal\Group	Reg Chow/dH_2_O	Reg Chow/Cu	Chol/dH_2_O	Chol/tap	Chol/Cu	Chol/Zn	Chol/Al
#1	none	none	none	none*	none*	none	none*
#2	none	none	none	none	none	focal/diffuse	none
#3	minimal	none	none*	focal/diffuse	focal/diffuse	none	minimal
#4	none	none	focal/diffuse	none	none	none*	none
#5						focal/diffuse	none

Total	0/4	0/4	1/4	1/4	1/4	2/5	0/5

* Sections showing patches unstained with lectin.

**Figure 2:**
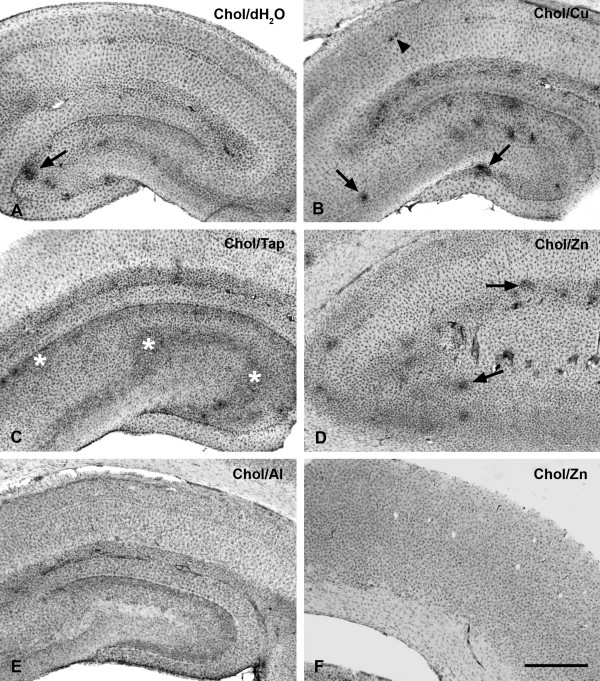
Lectin staining for microglia in the hippocampus (**A–E**) and cerebral cortex (**F**) of rabbits receiving cholesterol diets. The figure shows focal microglial activation evident as hyperintense spots (arrows in **A, B, D**), and a more diffuse pattern covering most the dentate hilus (asterisks in **C**). The arrowhead in panel **B **points to small spot of activated microglia in the CA1 pyramidal layer. Animals receiving drinking water supplemented with aluminum did not show significant microglial activation (**E**). None of the cholesterol-fed animals showed microglial activation in the cerebral cortex (**F**). Scale bar: 1,000 μm

**Figure 3:**
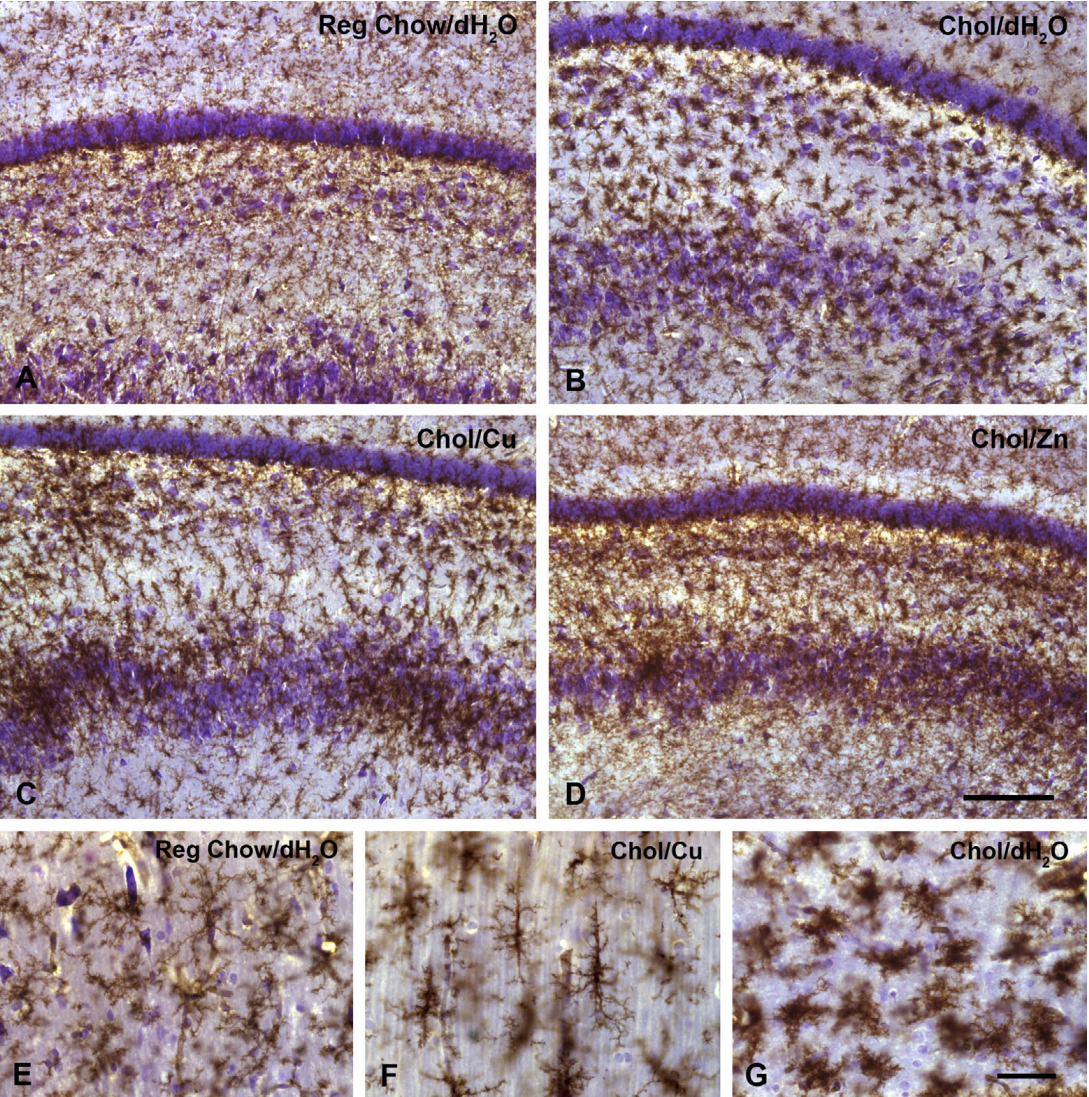
Comparison of resting and activated microglia in control and cholesterol-fed rabbits. **A–D**, dentate gyrus showing granule cell layer (near top) and hilus. Normal distribution of resting microglia in control rabbit (**A**) stands in contrast to activated microglia in hypercholesterolemic rabbits (**B–D**). Panel **B **shows diffuse distribution of activated microglia; panel **C **shows focal accumulations of activated microglia; panel **D **shows a combination of diffuse and focal patterns. **E–G**, high power views of various microglial morphologies, including resting cells (**E**), rod cells in stratum radiatum (**F**), and activated cells (**G**). Cresyl violet counterstain. Scale bars: 200 μm (A–D); 50 μm (E–G)

More pronounced microglial activation was observed in 5 out of the 22 animals that had been fed a cholesterol diet (Table 1). In these animals, microglial activation was evident by the presence of multiple foci of intensified lectin staining (e.g. Figs. 2A,B) and/or by a more diffuse presence of activated microglial cells throughout the dentate gyrus and the stratum lacunosum moleculare (Fig. 2C). Round spots of microglial activation measuring about 300–400 μm in diameter were most often seen in the hilus of the dentate gyrus (Figs. 2A,B, 3C,D). Their increased staining intensity was due to the accumulation of activated microglia displaying cell hypertrophy (Figs 3B,C,G). In one instance, very small foci of microglial activation could be observed in the stratum pyramidale (Fig. 2B). Animals that showed microglial activation also displayed conspicuous microglial rod cells, which were prominent in the stratum radiatum (Fig. 3F).

It is interesting to note that all animals, regardless of their diets, showed enhanced microglial staining in the subgranular zone clearly delineating the fascia dentata (Fig. 1A–C). This enhanced staining appeared to be due to a greater density of microglial cells in the subgranular layer, rather than to microglial activation as there was no evidence of cell hypertrophy. The stratum lacunosum moleculare was also well delineated by microglial staining in that cellular density there appeared to be slightly greater than elsewhere in the hippocampus (e.g. Figs. 1A–C). None of the animals demonstrated any evidence of microglial activation in the cerebral cortex overlying the hippocampal formation (Figs. 1D, 2F). Sections of the cerebral cortex revealed an even distribution of ramified microglia throughout.

**Figure 4:**
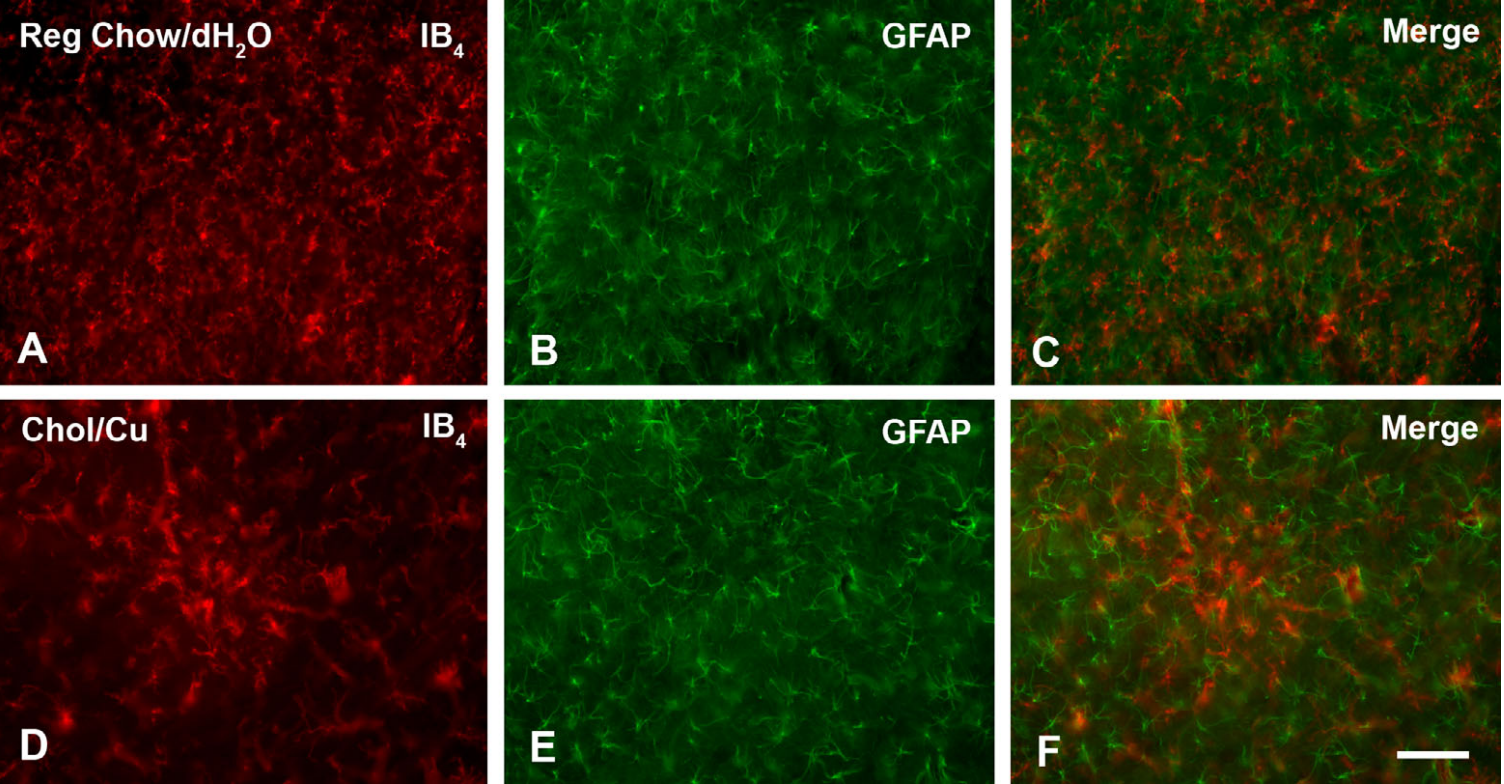
Double fluoresecent labeling for microglia with isolectin B_4 _and for astrocytes with anti-GFAP in rabbit hippocampus. **A–C**, uniform distributions of both glial cell types are evident in a control animal. **D–F**, focus of microglial activation in a cholesterol-fed rabbit shows normal staining pattern for astrocytes. Scale bar: 100 μm

When foci of microglial activation were examined at higher power, it was evident that the cells present in these areas were hypertrophied and their ramified processes retracted (Figs. 3B–D,G). Typically, these activated microglia were seen as round, focal formations within the hilus (Fig. 3C), but could also be seen to be distributed in a more widespread and diffuse fashion throughout the hilar gray and white matter strata (Figs. 3B,D). Microglial rod cells were frequently encountered in the stratum radiatum of those animals showing focal and diffuse activation patterns (Fig. 3F). Counterstaining with cresyl violet clearly revealed the neuronal layers of the hippocampal formation, but failed to show any evidence of neuronal damage or loss. In order to detect neurodegenerative changes, we also stained sections from cholesterol-fed animals that showed microglial activation for ubiquitin, but these studies failed to reveal any specific staining of neurons or their processes. To further analyze areas showing microglial activation we performed double fluorescent staining for both microglia and astrocytes, using a combination of lectin staining and GFAP immunostaining (Fig. 4). Included in these experiments were all five animals that had shown microglial activation in the hippocampus. Examination of double-stained preparations revealed that foci of microglial activation did not show concomitant increases in GFAP immunoreactivity (Figs. 4D–F). The intensity and distribution of GFAP immunoreactivity in these foci was no different from that observed elsewhere in the section or as seen in control animals (Figs. 4A–C), leading us to conclude that neuroinflammatory foci revealed by microglial activation were not subject to reactive astrogliosis.

**Figure 5:**
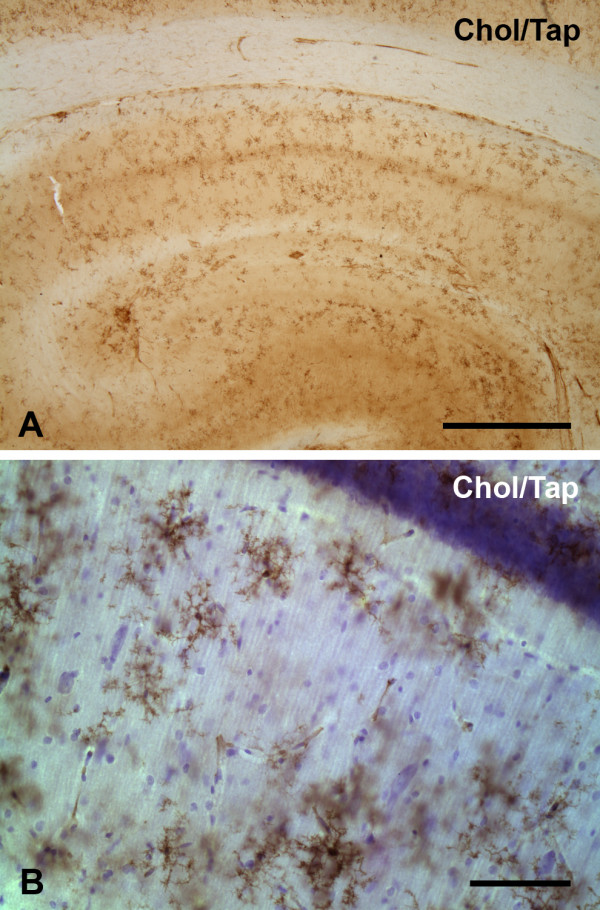
Patchy lectin staining of microglia throughout the hippocampus is shown at low power (**A**), and at high power with cresyl violet counterstaining (**B**). No evidence for microglial cell loss or other degenerative changes was detectable. Patchy staining is most likely an artifact of fixation. Scale bars: 1,000 μm (A); 100 μm (B).

A final observation that was evident in some of the cholesterol-fed animals, but not in controls, concerns the presence of microglia-free patches in lectin-stained sections (Table 1; Fig. 5). These patches are areas in any given section that are devoid of lectin staining, suggesting a localized loss of microglial cells. Shown in Fig. 5A is the most dramatic example of patchiness we were able to observe, and clearly the density of microglia in this particular section is much lower than what was normally seen in hippocampal sections (compare to Fig. 1). Examination of cell-free patches at high power did not reveal any signs of microglial cell death, and microglia in the vicinity of unstained patches were perfectly ramified and appeared normal and non-activated (Fig. 5B). No abnormalities could be detected in patchy areas using either cresyl violet staining or ubiquitin immunohistochemistry, and thus we attribute the spotty lectin staining to a tissue processing artifact, possibly related to fixation, rather than to a loss of microglial cells.

## Discussion

The current study serves to extend prior work from our and other laboratories regarding the sporadic neuroinflammation that occurs in hypercholesterolemic rabbits [5,20]. While on one hand confirming the intermittent nature of microglial activation and showing that it affected only 23% of all cholesterol-fed rabbits, compared to 30% in the study by Zatta et al. [20], the current findings also call attention to a previously unsuspected disconnection between increased amyloid production in hypercholesterolemic rabbits and microglial activation. There are at least four observations derived from the current study supporting the notion that increased amyloid production in hypercholesterolemic is not a direct stimulus for microglial activation. First is the mismatch between regions affected by increased amyloid immunoreactivity and neuroinflammation. Prior work has shown that the increase in amyloid induced by cholesterol occurs prominently in neuronal layers II, IV-V of the cerebral cortex, as well as in those of the hippocampus, including pyramidal and granule cell layers [3,4,15]. In contrast, the neuroinflammatory changes reported here are limited to the hippocampus with the dentate gyrus being affected to the greatest extent. Second, cholesterol-induced increases in intraneuronal amyloid immunoreactivity occur consistently in all hypercholesterolemic rabbits, while microglial activation occurs only in a relatively small fraction of these animals. Third, prior work has shown that supplementation of the drinking water with copper amplifies intraneuronal amyloid immunoreactivity, while addition of zinc does not [16]. This influence of metal ions over Alzheimer-like pathology is not mirrored by concomitant changes in neuroinflammation, as shown by our current results. In fact, it appears that neuroinflammation is most pronounced in animals that consumed zinc-supplemented drinking water. Fourth, we observed some, albeit minimal microglial activation in a control animal consuming standard rabbit chow and dH_2_O. Since control animals do not show intraneuronal amyloid immunoreactivity, the observed glial activation could not possibly represent a direct microglial response to amyloid. Although unexpected, this latter observation in a control animal serves to make an important point, namely, that studying microglial activation and distribution patterns is a very sensitive method for detecting subtle and localized changes in brain homeostasis that may not be detectable by other assays. Thus, microglia are indeed keen sensors of brain pathology [21]. This point is underscored further by our inability to uncover any evidence for neurodegenerative changes or neuronal loss in the five hypercholesterolemic rabbits that showed pronounced microglial activation. Neither counterstaining with cresyl violet nor ubiquitin immunolabeling revealed any neuronal abnormalities. In addition, there was a striking absence of reactive astrocytes in those focal areas demonstrating microglial activation, which leads us to think that the disturbance that triggered microglial activation was not sufficiently severe to cause serious neuronal damage and subsequent astroglial scarring.

There are numerous reports, most of them *in vitro*, describing how amyloid peptides stimulate detrimental microglial activation (e.g. [12,14,22,23]). These *in vitro *studies have been critical for supporting the notion that presence of amyloid plaques in AD brain leads to a chronic neuroinflammatory response, which many believe plays a central role in the development of Alzheimer's disease [6,8,10,11,24-27]. Our current findings in cholesterol-fed rabbits do not offer additional support for the idea that amyloid directly triggers neuroinflammation, as already explained. However, it is important to point out that most of the amyloid accumulation in hypercholesterolemic rabbits is intraneuronal and that deposition of amyloid in the extracellular space occurs only rarely [5,15,20]. This, of course, could mean that most of the intracellular amyloid never reaches microglial cells surveying the extracellular milieu. As far as the ability of copper in the drinking water (but not zinc or aluminum) to amplify cholesterol-induced amyloid accumulation, we hypothesize that this is due to copper's unique ability to inhibit amyloid clearance from brain [28].

So what might be the nature of an underlying perturbation that triggers the sporadic and localized neuroinflammatory reactions observed? One possibility that comes to mind is vascular inflammation and an associated breach in the blood brain barrier (BBB). Previous studies in the hypercholesterolemic rabbits have shown leakage of Evans Blue dye into the brain parenchyma, as well as increased vascular immunoreactivity with MECA-32 [1], an antibody which recognizes an endothelial cell epitope that is downregulated as the BBB matures during development [29]. Reexpression of the MECA-32 antigen has been found to occur during experimentally induced neuroinflammation [30]. Thus, high levels of serum cholesterol in rabbits may induce vascular changes similar to early inflammatory lesions of atherosclerosis, and this vascular inflammation may trigger microglial activation. However, in other animal models, an induction of peripheral inflammation and increased BBB permeability associated with extravasation of serum proteins has been shown to occur without reactive microgliosis or astrogliosis [31]. Thus, further studies focused specifically on examining the relationship of vascular inflammation and microglial activation in hypercholesterolemic rabbits seem to be indicated.

A final consideration pertains to the basic understanding of the functional significance of microglial activation and neuroinflammation, i.e. whether it is beneficial or harmful. Given the great abundance of microglial cells throughout the CNS, as shown in the micrographs presented here, it is difficult to see an evolutionary advantage in having this many potentially dangerous immune effector cells populate an organ that is relatively incapable of regeneration. In our view, the only way to reconcile microglial abundance with an evolutionary advantage is to accept that these cells are constitutively neuroprotective, and that the spatially restricted microglial activation observed here is a reflection of an ongoing rescue effort [32,33]. In other words, microglia get activated when neurons get damaged, rather than the other way around. Thus, we believe that the current findings demonstrating focal microglial activation in the hippocampus are a reflection of focal neuronal damage, which is likely to be minor since it is not demonstrable with routine histological stains, with specific markers of neurodegeneration, or with markers of astrogliosis. The increased and sporadic occurrence of microglial activation in rabbits on cholesterol diets suggests that dietary factors can directly affect the hippocampus.

## Conclusion

The current histopathological analysis underscores the extreme sensitivity of microglial reactions – they are truly biological sensors of neuropathology. The sporadic and focal nature of the microglial activation observed in hypercholesterolemic rabbits suggests that any damage inflicted on hippocampal neurons is very slight, and potentially reversible. We suspect that high-cholesterol diets, which are very atypical for rabbits and rodents in general, are sufficiently adverse to upset the metabolism of some neurons in some animals to trigger a microglial response. By analogy, it now seems reasonable to think that dietary factors in humans may subtly influence brain homeostasis, and that diet-induced disturbances are demonstrable through analysis of microglia during post-mortem examination.

## Competing interests

The author(s) declare that they have no competing interests.

## Authors' contributions

QX carried out the histopathological studies and drafted the manuscript. DS initiated this collaborative study. WS participated in analysis of histopathological findings and design of figures. All authors completed the final version of the manuscript.
